# Combatting renal ciliopathies

**DOI:** 10.1186/2046-2530-1-S1-O30

**Published:** 2012-11-16

**Authors:** RH Giles

**Affiliations:** 1 Dept. Nephrology, University Medical Center Utrecht, the Netherlands

## 

A hallmark of many ciliopathies are renal cysts, ultimately disrupting kidney architecture and resulting in end-stage renal disease as the most common cause of mortality. Yet despite being the largest demand for renal replacement therapy (ie. dialysis, kidney transplantation) in young patients, the exact etiology of nephronophthisis (NPHP) and polycystic kidney disease is still largely unknown. Many gene products associated with NPHP and related disorders do not directly affect cilia, but appear to be important in tubular architecture, lumen formation, and polarized exocytosis. Personalized therapy to delay disease progression is beginning to explore gene replacement therapy, through use -for example- of induced pluripotent stem cells (iPSCs). For patients with nonsense mutations in ciliopathy genes, read-through drugs such as PTC124 and modified aminoglycosides might hold future promise. Furthermore, several drug screens in cell-based, zebrafish, and murine models have suggested new pathways preserving kidney function. Although much of this work has yet to be validated in human patients, this overview will discuss the approaches currently being employed in the context of improving ciliopathy treatment options.

